# Analysis of Volatile Compounds in Processed Cream Cheese Models for the Prediction of “Fresh Cream” Aroma Perception

**DOI:** 10.3390/molecules28207224

**Published:** 2023-10-23

**Authors:** Coline Caille, Mariem Boukraâ, Cécile Rannou, Angélique Villière, Clément Catanéo, Laurent Lethuaut, Araceli Lagadec-Marquez, Julia Bechaux, Carole Prost

**Affiliations:** 1 Oniris—UMR CNRS 6144 GEPEA—MA(PS)2/USC INRAE 1498 TRANSFORM, 44322 Nantes, France; mariem.boukraa@uliege.be (M.B.); cecile.rannou@oniris-nantes.fr (C.R.); clement.cataneo@oniris-nantes.fr (C.C.); laurent.lethuaut@oniris-nantes.fr (L.L.); 2 Bel Group—Bio-Engineering Team, 41100 Vendôme, France

**Keywords:** processed cream cheese models, ĸ-carrageenan, agar-agar, volatile compound release, sensory analysis, rate all that apply, prediction, machine learning, random forest

## Abstract

Controlling flavor perception by analyzing volatile and taste compounds is a key challenge for food industries, as flavor is the result of a complex mix of components. Machine-learning methodologies are already used to predict odor perception, but they are used to a lesser extent to predict aroma perception. The objectives of this work were, for the processed cream cheese models studied, to (1) analyze the impact of the composition and process on the sensory perception and VOC release and (2) predict “fresh cream” aroma perception from the VOC characteristics. Sixteen processed cream cheese models were produced according to a three-factor experimental design: the texturing agent type (κ-carrageenan, agar-agar) and level and the heating time. A R-A-T-A test on 59 consumers was carried out to describe the sensory perception of the cheese models. VOC release from the cheese model boli during swallowing was investigated with an in vitro masticator (Oniris device patent), followed by HS-SPME-GC-(ToF)MS analysis. Regression trees and random forests were used to predict “fresh cream” aroma perception, i.e., one of the main drivers of liking of processed cheeses, from the VOC release during swallowing. Agar-agar cheese models were perceived as having a “milk” odor and favored the release of a greater number of VOCs; κ-carrageenan samples were perceived as having a “granular” and “brittle” texture and a “salty” and “sour” taste and displayed a VOC retention capacity. Heating induced firmer cheese models and promoted Maillard VOCs responsible for “cooked” and “chemical” aroma perceptions. Octa-3,5-dien-2-one and octane-2,3-dione were the two main VOCs that contributed positively to the “fresh cream” aroma perception. Thus, regression trees and random forests are powerful statistical tools to provide a first insight into predicting the aroma of cheese models based on VOC characteristics.

## 1. Introduction

Texture and flavor are determinant factors in product liking and consumer acceptance [1,2]. The desirable texture characteristics of processed cheeses are “smooth”, “spreadable” and “melting in the mouth”, and the typical flavor notes are “fresh cream”, “cheese”, “acid” and slightly “salty” [3,4,5]. Among these characteristics, “fresh cream” is one of the main drivers of the aroma of processed cream cheeses. Some researchers modified food texture and/or flavor by changing the composition, such as the fat or protein type or content, or by adding texturing agents [6,7]. Investigating the behavior of texturing agents with different ionic charges is of great interest, as a wide range of hydrocolloids can be used in dairy products. Carrageenans are among the most widely used texturing agent in the dairy industry, as their negative sulfate charges electrostatically interact with the positive charges of casein micelles and whey proteins, allowing the formation of stable products [8,9]. Agar-agar may be considered as a type of carrageenan with fewer sulfate groups, and therefore, it has fewer negative charges [10,11,12,13]. In addition to composition changes, cheese texture and flavor can be modified by changes in the process, such as the mixing rate and duration or the heating temperature [4,14,15]. Maillard and creaming reactions during heating can indeed affect the texture and flavor of cheeses [16]. Some researchers successfully carried out experimental designs to analyze the impact of composition or process factors on the characteristics of dairy products, such as processed cheeses or yogurts [14,17].

The interactions between texture and flavor release and the perception of dairy products have been largely investigated in the last decades. Increasing the hardness or firmness of foods generally leads to a reduction in the perception of flavor. The interactions between texture and flavor may depend on the type of the flavor compound but also on the type of texturing agent [2,6]. In order to get rid of these interactions between texture and flavor, researchers have worked on iso-texture products [18,19]. While texture is fairly easy to quantify, flavor is less so. Indeed, flavor is a complex mix of components (aroma, taste, trigeminal sensations) that interact with each other. The interactions between these parameters, combined with the different perceptions and preferences of the consumers, make flavor a characteristic that is difficult to quantify objectively [20]. Moreover, flavor is matrix-dependent and can hardly be generalized. However, it is possible to study flavor using sensory and instrumental analyses and then to study the statistical links between these data [21]. The release and perception of flavor compounds occur during the mastication process; the swallowing point attracts special attention, as it is known to be a key stage for Volatile Organic Compound (VOC) release and aroma perception [22].

To quantify flavor and texture perceptions, Quantitative Descriptive Analysis (QDA) is a widely used descriptive sensory test involving trained panelists [21,23]. As this analysis can be time-consuming and costly, new sensory methodologies involving untrained panelists have been developed, such as the Rate-All-That-Apply (R-A-T-A) method [24,25]. The R-A-T-A test is an intensity-based Check-All-That-Apply (C-A-T-A) variant. It consists of ticking, in a predefined list, descriptors that are relevant to describe a product and then scoring the intensity of the descriptors ticked. In addition to its originality, R-A-T-A has the advantage of evaluating a high number of products and sensory descriptors in a short time. Thus, this methodology seems suitable for describing both the flavor and texture sensory characteristics of cheese models.

Different instrumental analyses have been developed to study VOC release during mastication. Nosespace methods involve the continuous analysis of the expired air of the panelists, or the air leaving a masticator, while food is chewed. The VOCs are analyzed with an Atmospheric Pressure Chemical Ionization (APCI) or a Proton Transfer Reaction (PTR)–Mass Spectrometry (MS) apparatus [2,6]. A disadvantage of these methods is that they only allow the monitoring of a limited number of VOCs, known in advance and often added during an aromatization step. Another technique involves a Tenax adsorbent or a Solid-Phase Microextraction (SPME) fiber, which is placed in the mouth or the nose of panelists or at the end of a masticator [26]. In addition, another method consists of transferring in vivo or in vitro boli collected in the mastication stage to vials and analyzing the VOCs by headspace (HS)-SPME, followed by gas chromatography (GC) coupled with mass spectrometry (MS) [27]. These last methods have the advantage, with the use of SPME in particular, of enabling both the identification and semi-quantification of the totality of endogenous VOCs of the chewed product. The detection of VOCs present at trace levels or with subtle peak area differences in complex matrices such as cheeses may be difficult while working with a single-quadrupole mass spectrometer. Thus, coupling HS-SPME with gas chromatography time-of-flight MS ((ToF)MS) is of great interest [28,29]. Indeed, due to its high acquisition frequency, this type of detector increases signal accuracy, which in turn facilitates deconvolution. Deconvolution is a mathematical algorithm used to find the real VOCs when they are not well resolved on the chromatogram (co-elution, very low concentrations).

Several statistical tools have been developed to study the relationship between sensory description data and instrumental VOC release. The more classical methods are correlation analysis or Partial Least-Squares (PLS) regression and have already been used to predict the aroma of dairy products [4,21,30,31]. A more original method is the use of a machine-learning algorithm, such as regression trees (RTs) and random forests (RFs) [32,33]. One of the main advantages of RFs is the robustness of the prediction, which is based on the principle of bagging (bootstrap aggregating). Indeed, each decision tree is created by a double-randomization process: a bootstrap of the products and a random selection of the best VOCs among a third of all compounds at each node of the tree. Moreover, unlike PLS regression, machine-learning algorithms require fewer assumptions (e.g., normality, absence of collinearity) for modeling to be correct [34]. While RT and RF methodologies have already been used to predict aroma sensory descriptors from the VOC profile of sweet pepper [20], they have never been used, to our knowledge, to predict aroma sensory descriptors from the VOC profile of in vitro cheese boli. Thus, a novelty of this study was the use of the random forest methodology to investigate the contribution of the VOCs released during swallowing from in vitro cheese boli to the aroma perception.

The objectives of the present work were, for the processed cream cheese models studied, (1) to analyze the impact of texturing agents and heating on texture, odor and flavor perceptions and on the release of VOCs at the swallowing point and (2) to predict “fresh cream” aroma sensory perception from the VOC release during swallowing. Thus, the present study will provide a deeper understanding of the impact of composition and laboratory-scale process factors on sensory perception and VOC release while eating. It will demonstrate the efficiency of the regression tree and random forest methods to predict aroma perception from instrumental VOC data. Moreover, this work will provide a better comprehension of the key VOCs of the processed cream cheese models that contribute to the “fresh cream” aroma sensory perception.

**Figure 1:**
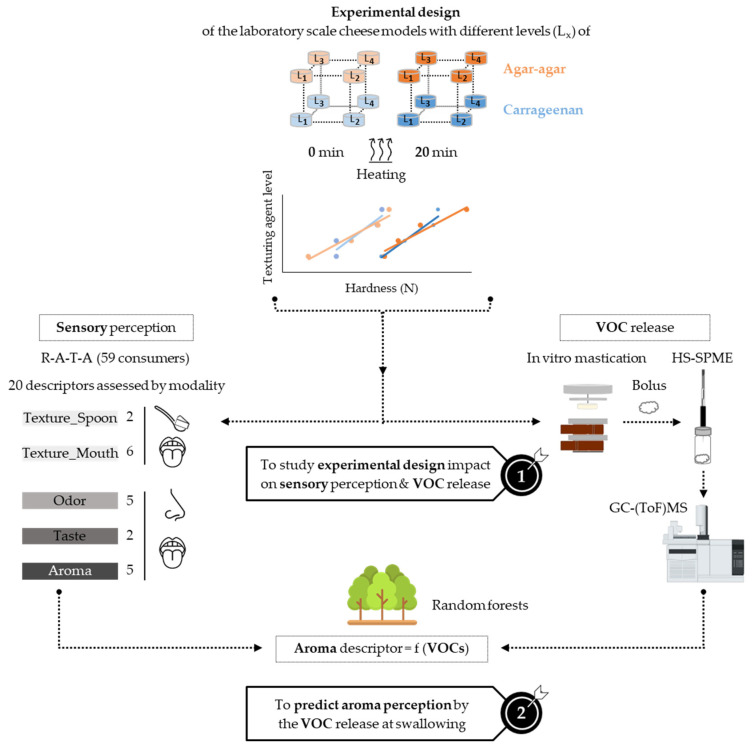
Diagram of the scientific approach.

The scientific approach of the present work is illustrated in Figure 1. Firstly, processed cream cheese models were produced at the laboratory scale according to a three-factor experimental design. The factors and their levels were selected to act as levers for creating clear variability in texture and flavor between the processed cream cheese models. Two composition factors were selected: the texturing agent type (κ-carrageenan, agar-agar) and the texturing agent level (four levels). These two texturing agents were studied because, as they have different chemical structures and thus different ionic charges, we hypothesize that they will have different retention capacities for volatile compounds. The four levels of the texturing agent were determined to obtain iso-hardness products regardless of the texturing agent type, i.e., so that the processed cream cheese models had comparable increases in hardness regardless of the texturing agent type (Figure 1). We hypothesize that working with cheese models with the same hardness, regardless of the texturing agent, will allow a better understanding of the behavior of the two texturing agents and that it will enable the focus to be placed on other properties of the products, such as the odor and the flavor. In addition to the two composition factors, a third one, related to the laboratory-scale process, was selected: the heating time. As we hypothesized that heating would have an impact on flavor release and perception, unheated and heated processed cream cheese models were produced. An innovative aspect of this study was that κ-carrageenan and agar-agar cheese models had similar hardness increases with heating. Secondly, sensory and instrumental analyses were performed on the cheese models from the experimental design. The products were subjected to a sensory description, particularly that of their aroma, by performing a R-A-T-A test. In parallel with the sensory analysis, the cheese models were destructured using an in vitro masticator (Oniris, 2013, device patent No. 1355509) until the swallowing point. The resulting in vitro boli were sampled in vials; the VOCs were then extracted by HS-SPME and analyzed by gas chromatography coupled to a time-of-flight mass spectrometer. Untargeted VOC analysis was performed, and the specific ion peak areas of each VOC identified were used to carry out a comparative approach between the cheese models. Finally, current and adapted machine-learning methodologies (regression trees and random forests) were carried out to predict the “fresh cream” aroma perception from the VOC release during swallowing, a novel step in aroma prediction.

## 2. Results and Discussion

### 2.1. Laboratory-Scale Production of Cheese Models with the Same Hardness, Regardless of the Texturing Agent Type

The processed cream cheese models in the present work were produced at the laboratory scale according to the experimental design displayed in Table 1.

The results of a three-way ANOVA on instrumental texture data showed that second-order interactions were not significant (*p* > 0.05). The effect of each factor studied was therefore independent of the level of variation in the other factors.

**Figure 2:**
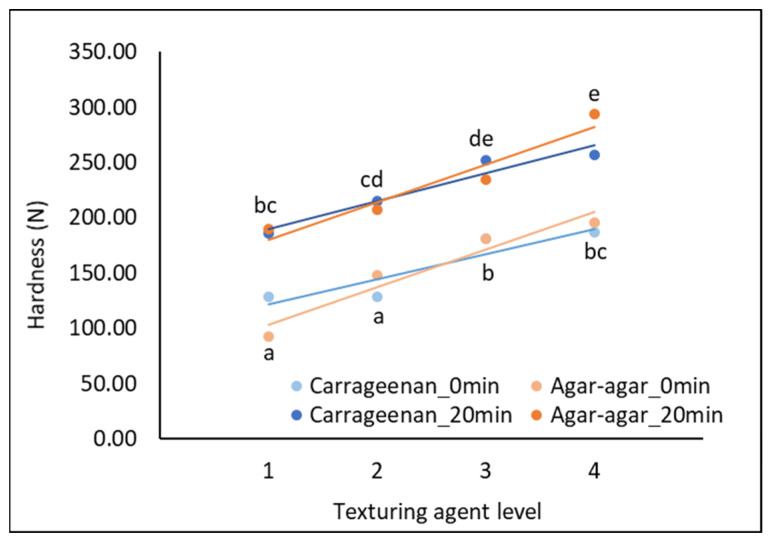
Hardness evolution (instrumental data) of the initial processed cream cheese models as a function of the texturing agent level. The different letters indicate statistically significant differences (Least Significant Difference method, α = 5%); texturing agent type: carrageenan, agar-agar; for carrageenan cheese models: 1 = 0.20% (*w*/*w*), 2 = 0.35% (*w*/*w*), 3 = 0.50% (*w*/*w*), 4 = 0.65% (*w*/*w*); for agar-agar cheese models: 1 = 0.60% (*w*/*w*), 2 = 0.90% (*w*/*w*), 3 = 1.20% (*w*/*w*), 4 = 1.50% (*w*/*w*).

Heating the samples for 20 min significantly increased the hardness of the processed cream cheese models (F = 87.56, *p* = 0.0000; Figure 2). The increase in hardness during the heating step of cheese manufacture has already been reported [5,16]. It has been attributed to the creaming reaction, a well-known phenomenon in the processed cheese industry. Lee et al. [16] showed that the creaming reaction was mainly due to protein interactions. Indeed, these researchers observed that heating caused changes in the protein networks in the cheeses studied, leading to an increase in viscosity up to a maximum, which was reached after 25 min of heating; a decrease in viscosity was then observed due to the collapse of the protein network. The observed changes in the protein network could be explained by the denaturation of milk proteins, which occurs at around 75 °C [4]. Due to the remarkable textural changes occurring during the heating step, some scientists have referred to it as a texturization step [5]. The increase in hardness during heating could also be due to structural changes in the milk fat fraction. Indeed, Pluta-Kubica et al. [35] reported that longer melt holding times led to a decrease in the size of milk fat droplets and thus to an increase in the complex modulus G*, i.e., the rigidity of model cheeses. In addition, the structure of the texturing agents added to the cheese models studied in this work could also be modified with heating. In fact, the gelation temperature of κ-carrageenans is between 35 and 65 °C, and agar-agar forms a gel at 30–35 °C [10,36]. Therefore, structural changes in both the initial cheese model components (proteins, fat) and texturing agents could explain the increase in hardness observed upon heating for 20 min.

A “texturing agent level” effect on hardness was noted (F = 24.14, *p* = 0.0000). As expected, the higher the texturing agent level, the harder the cheese models were.

No “texturing agent type” effect on hardness was observed (F = 0.07, *p* = 0.7939). This result confirmed that the objective of producing laboratory-scale processed cream cheese models with the same hardness (instrumentally measured), regardless of the texturing agent type, was successfully achieved for both unheated and heated samples (Figure 2). In this way, sensory and instrumental (in vitro mastication coupled with HS-SPME-GC-(ToF)MS) analyses could be performed on the processed cream cheese models without bias due to differences in hardness between the two texturing agents.

### 2.2. Impact of the Processed Cream Cheese Model Composition and Process on the Sensory Perception

The results for the “product” factor of a two-way ANOVA performed on R-A-T-A data are displayed in Table 2. All of the sensory descriptors of texture and taste presented significant differences between the processed cream cheese models (*p* < 0.05). Concerning the aroma descriptors, three out of five were significant: “fresh cream” (*p* = 0.0000 ***), “fresh cheese” (*p* = 0.0000 ***) and “chemical” (*p* = 0.0000 ***). Only one odor descriptor out of five was significant: “fermented” (*p* = 0.0392 *). The cheese models were well discriminated according to their texture perception, which is consistent with the instrumental texture measurements. Flavor differences were perceived regarding both taste and aroma. Unfortunately, as can be expected when working with such products, the odor perception did not allow good discrimination between the products.

**Figure 3:**
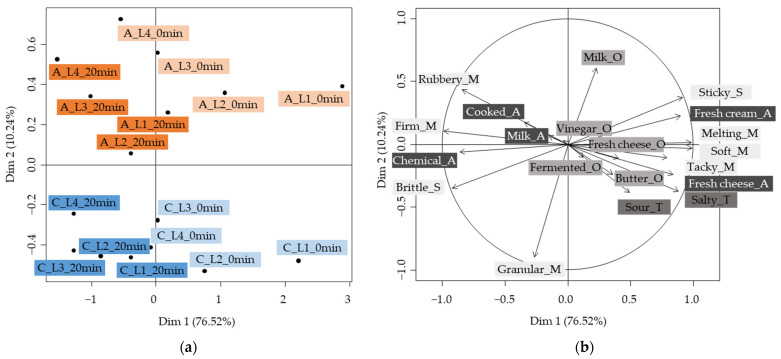
Results of a CVA performed on the R-A-T-A data. (**a**) Product map. A: agar-agar samples (orange shading); C: carrageenan samples (blue shading); LX: level X of texturing agent; heating time: 0 min (light shading)–20 min (dark shading). (**b**) Correlation circle of the sensory descriptors. Shading—very light gray: texture descriptors (M: mouth; S: spoon); light gray: odor descriptors (O); dark gray: taste descriptors (T); very dark gray: aroma descriptors (A).

Figure 3a illustrates the cheese map and the sensory descriptor correlation circle from a Canonical Variate Analysis (CVA) performed on R-A-T-A data. A CVA was performed rather than a PCA because CVA is a well-adapted approach when dealing with sensory profiling data [37]. Indeed, it takes into account the panelist variability by analyzing the entire set of sensory data and not only the means of the different descriptors of each product. In addition, the product map from the CVA maximizes product discrimination while minimizing subject effects for the same product.

The first two components explained 86.76% of the total variance in the data. The first component, Dim 1, explained 76.52% of the variance and revealed a “heating” effect. Indeed, Dim 1 separated the unheated samples (Dim 1 highest values) from the heated (Dim 1 lowest values) samples. The second component, Dim 2, explained 10.24% of the variance and showed a “texturing agent type” effect. Indeed, Dim 2 separated the carrageenan cheese models (Dim 2 lowest values) from the agar-agar cheese models (Dim 2 highest values). Within the undersquare of the CVA product map, a “texturing agent level” effect can be noticed: from right to left, the cheese models were positioned from the lowest to the highest texturing agent level.

Figure 3b presents the correlation circle of the sensory descriptors. The descriptors related to the unheated samples, i.e., those that contributed positively to the first component, were “sticky”, “melting”, “soft” and “tacky” for the texture descriptors; “fresh cream” and “fresh cheese” for the aroma ones; and “salty” for the taste descriptor. The descriptors associated with the heated samples, i.e., those that contributed negatively to the first component, were “firm” and “brittle” for the texture descriptors and “chemical” and “cooked” for the aroma descriptors. The cheese models with the same instrumental hardness were perceived to have the same sensory “firmness”. This result showed that the aim of obtaining cheese models with the same hardness, regardless of the texturing agent type, was successfully achieved.

The heating step, which lasted 20 min, could have resulted in the denaturation of milk proteins, a decrease in the fat droplet size and the activation of the gelling properties of the texturing agents [4,10,35], which could thus explain the observed increase in hardness (Figure 2). This change in texture could explain the increase in “firm_m” and “brittle_s” sensory perceptions. This result is consistent with that of Kohama-Kubouchi et al. [4] for cheeses, who found that “softness” and “melting in the mouth” perceptions decreased with higher heating temperatures. In our study, the decrease in typical cheese model aroma notes such as “fresh cream” and “fresh cheese” and taste notes such as “sour” and “salty” with heating could also be attributed to the cheese model hardness increase. Indeed, some researchers have already noted strong interactions between texture and flavor perceptions of dairy products. In general, firmer products were perceived as having less flavor [2]. Kohama-Kubouchi et al. [4] observed that “yogurt aroma”, “acetic aroma” and “acidity” perceptions decreased significantly with higher mixing temperature, i.e., with firmer cheeses. Studying pectin and gelatin gels, Boland et al. [38] also observed an increase in thickness and a decrease in odor and flavor perceptions with increasing rigidity. In addition, Saint-Eve et al. [39] noticed a decrease in aroma perception with the increase in yogurt viscosity, which is consistent with our findings. However, Saint-Eve et al. [40] found that the salty perception of model cheeses was not influenced by the textural properties. This last result on taste perception differs from our findings; this may be due to the fact that the texture variations were not the same as those in our study. In parallel with the increase in hardness during heating, the Maillard reaction generates new VOCs, such as furans, pyrazines or sulfur compounds [28,41], which could explain the increase in the perception of “cooked” and “chemical” aromas. These results are in good agreement with those of Jo et al. [42], who observed that ultra-pasteurized milk subjected to higher heat treatment than high-temperature-short-time (HTST) pasteurized milk was perceived to have “cooked” and “sulfur” flavor notes compared to HTST milk. The intensity of the heat treatment should be carefully controlled as “overcooked” notes could be perceived at temperatures above 120 °C [43].

Considering texture descriptors other than “firmness”, although the hardness of the processed cream cheese models was comparable regardless of the texturing agent type (Figure 2), the κ-carrageenan cheese models were perceived as “granular” and “brittle”, whereas the agar-agar cheese models were perceived as “rubbery” (Figure 3b). These results are in line with other scientific findings, as ĸ-carrageenan is known to form gels with a grainy and brittle texture, whereas agar-agar is known to form rigid gels [36,44,45]. Concerning the taste descriptors, κ-carrageenan cheese models were perceived as more “sour” and “salty” than agar-agar products. Marshall et al. [18] found that the perception of taste, such as “sweetness”, was more intense in carrageenan gels than in other hydrocolloid gels. This result, as with ours, seems to suggest that products containing carrageenan would be perceived with more taste. The improved taste perception of κ-carrageenan products could be attributed to the fact that this texturing agent produced brittle gels with faster destructuring and larger surface area upon chewing, resulting in a greater release of taste compounds [45]. From an odor point of view, agar-agar cheese models were perceived to have more milky notes than κ-carrageenan products. According to previous studies, agar-agar seemed to have a lower retention capacity than carrageenan for certain compounds, including VOCs [46,47,48]. Volatile compounds could be more released and, therefore, more perceived in the presence of agar-agar compared to carrageenan, which could explain the milk odor of processed cream cheese models containing agar-agar. This result differs from that of Chai et al. [49], who observed that the perceived intensity of an “orange” aroma, given comparable gel firmness, was lower in an agar-agar gel than in a κ-carrageenan gel. The perception of odor and aroma differences between these two texturing agents might depend on the product or the sensory descriptor analyzed.

Thus, unheated samples were perceived to have a “melting”, “soft” and “tacky” texture and typical soft-cheese aroma notes, such as “fresh cream” and “fresh cheese”. The addition of a heating step generated cheese models that were perceived as having a “firm” texture and a “cooked” and “chemical” aroma. In terms of the texturing agent type, κ-carrageenan cheese models were perceived as having a “granular” and “brittle” texture, and a “sour” and “salty” taste. Agar-agar cheese models were perceived as having a “rubbery” texture and “milk” odor notes. In this way, the factors of the experimental design seemed to have an impact on the sensory perception, whether in terms of texture, odor or flavor.

### 2.3. Release of Volatile Compounds

#### 2.3.1. VOCs from the Processed Cream Cheese Models

A VOC analysis of the processed cream cheese boli at the swallowing point (in vitro destructuring followed by HS-SPME) resulted in 200 VOCs being (tentatively) identified (Table 3). They belonged to different chemical classes commonly found in cheeses: alkanes (30 VOCs, i.e., 15% of the total number of VOCs), aldehydes (24 VOCs, i.e., 12%), acids (21 VOCs, i.e., 11%), methyl ketones (20 VOCs, i.e., 10%), alcohols (16 VOCs, i.e., 8%), alkenes (16 VOCs, i.e., 8%), ketones (12 VOCs, i.e., 6%), furans (12 VOCs, i.e., 6%), lactones (11 VOCs, i.e., 5%), aromatic hydrocarbons (11 VOCs, i.e., 5%), nitrogen compounds (10 VOCs, i.e., 5%), esters (7 VOCs, i.e., 4%), sulfur compounds (6 VOCs, i.e., 3%) and terpenes (4 VOCs, i.e., 2%). Similar chemical classes were found by Kohama-Kubouchi et al. [4] and Ningtyas et al. [50] when investigating cheese VOCs by HS-SPME. In terms of the number of VOCs, the main chemical classes in the present study were alkanes (30 VOCs), aldehydes (24 VOCs), acids (21 VOCs) and methyl ketones (20 VOCs). Bertrand et al. [28] identified a high number of alkanes (HS-SPME-GCxGC-(ToF)MS) in a study on similar types of cheeses, i.e., processed cheeses, which is coherent with our results. During heating, lipid fatty acid oxidation and amino acid fragmentation can lead to the formation of alkanes [28]. In addition, the use of a time-of-flight mass spectrometer, followed by deconvolution of the signal obtained, enabled the extensive mapping of VOCs, which could explain the identification of many alkanes and alkenes in this study. Moreover, Jeon et al. [51] observed that the acid group was the chemical class with the highest number of VOCs (HS-SPME) from cream cheeses. Thus, many alkanes and acids seem to be present in soft cheeses.

#### 2.3.2. Impact of the Processed Cream Cheese Model Composition and Process on the VOC Release during Swallowing

**Figure 4:**
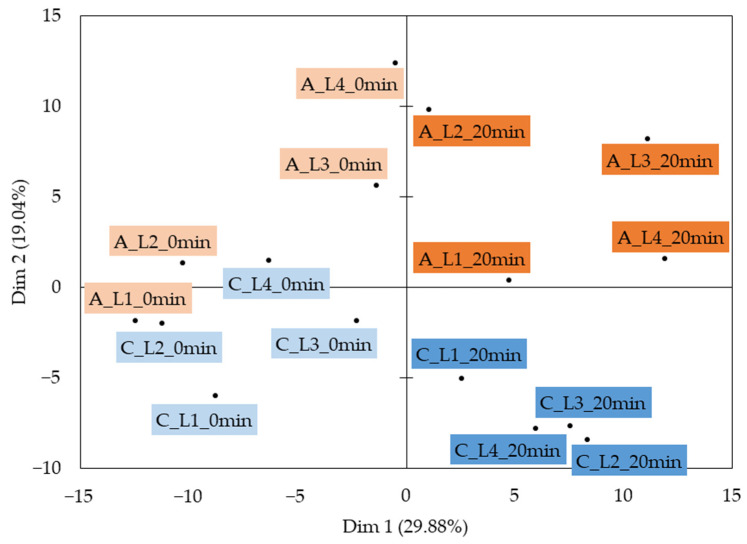
Product map of the standardized PCA performed on the ion peak areas of the VOCs of the cheese model in vitro boli. A: agar-agar samples (orange shading); C: carrageenan samples (blue shading); LX: level X of texturing agent; heating time: 0 min (light shading)–20 min (dark shading).

Figure 4 illustrates the cheese model in vitro bolus map of the standardized Principal Component Analysis (PCA) performed on the specific ion peak areas of the VOCs of the samples. The first two components explained 48.92% of the total data variance. The first component, Dim 1, explained 29.88% of the variance and revealed a “heating” effect. Indeed, Dim 1 separated the unheated (Dim 1 lowest values) from the heated (Dim 1 highest values) samples. The second component, Dim 2, explained 19.04% of the variance and showed a “texturing agent type” effect. Indeed, Dim 2 separated the carrageenan cheese model boli (Dim 2 lowest values) from the agar-agar cheese model boli (Dim 2 highest values). Within the undersquare of the PCA product map, a “texturing agent level” effect could be detected: from left to right, the cheese model boli were placed, to a certain extent, from the lowest to the highest texturing agent level.

The VOCs associated with the unheated samples, i.e., those that contributed negatively to the first component, were mainly lactones (γ-hexalactone, δ-octalactone, γ-octalactone, δ-hexalactone), some methyl ketones (acetophenone, octa-3,5-dien-2-one), diketones (octane-2,3-dione, butane-2,3-dione) and acids (benzoic acid, acetic acid, butanoic acid, pentanoic acid) (Table 3 and Table 4). Lactones and ketones are naturally present in milk [52]; it is thus consistent to observe these chemical classes in the unheated samples. In this study, butane-2,3-dione, a highly volatile compound known for its strong buttery odor [53], was associated with the unheated samples. This could be due to the fact that this VOC, naturally present in cheeses [54], has a low molecular weight (86.09 g·mol^−1^) and boiling point (87.50 °C) [55]. Butane-2,3-dione could thus evaporate, and its content decreased upon heating for 20 min, which could explain its decreased release with thermal treatment. It is worth noting that the butane-2,3-dione content in cheeses seemed to depend on the intensity of the thermal treatment (both temperature and duration). Indeed, while studying the impact of the storage temperature on the development of VOCs in cheeses, Sunesen et al. [56] noticed that the two VOCs most negatively influenced by an increase in storage temperature from 5 to 37 °C were 3-hydroxybutan-2-one and its oxidized form butane-2,3-dione. However, Bertrand et al. [28] observed an increase in butane-2,3-dione in a model cheese during cooking up to 120 °C and then a decrease in the level of this compound at the highest temperatures. Cerny [57] and Parker [58] also found that butane-2,3-dione was an odor-active compound from the Maillard reaction. Moreover, Zhang et al. [59] noted a decrease in organic acid content with milk heating, which is consistent with the fact that the unheated cheese models of this study contained more acids such as benzoic acid, acetic acid, butanoic acid and pentanoic acid compared to the heated samples.

The VOCs related to the heated samples, i.e., those that contributed positively to the first component, were mainly sulfur compounds (S-methyl ethanethioate, 1,3-thiazole, 2-methylthiophene), aldehydes (2-methylbutanal, (E)-but-2-enal, (E)-pent-2-enal), furans (2-methylfuran), aromatic hydrocarbons (benzene) and some methyl ketones (hexan-2-one, pentan-2-one, heptan-2-one) (Table 3 and Table 4). During heating, the Maillard reaction is the main reaction responsible for flavor generation [58]. While this reaction does not necessitate high temperatures, the formation of flavor compounds occurs at cooking temperatures [60]. The Maillard reaction generates a wide range of VOCs, such as sulfurs (thiazoles, thiophenes) [28,57], furans [41] and Strecker aldehydes such as 2-methylbutanal [43,54,60], which compare well with our results. In addition, methyl ketones are naturally present in milk, but lipid degradation during heating can enhance their formation [61], which is in good agreement with the present findings. Zhang et al. [59] also observed an increase in the amounts of methyl ketones with heat treatment, especially heptan-2-one. The origin of aromatic hydrocarbon compounds such as benzene in dairy products has not been clearly identified [61].

Although the hardness of the processed cream cheese models was identical regardless of the texturing agent type (Figure 2), differences in the VOC release between the carrageenan and the agar-agar cheese models were observed. The VOCs related to agar-agar cheese model boli, i.e., those that contributed positively to the second component, were mainly alkanes (4-methyloctane, 2,4-dimethylheptane, 2,3-dimethylheptane, 3-methylheptane, 4-methylheptane, 2,5-dimethylhexane, 2,4-dimethylhexane, 1,1,3-trimethylcyclopentane, undecane, 2-methylheptane, decane, 3-methylpentane), alkenes (5-ethyl-2,4-dimethylhept-2-ene, 2,4-dimethyldec-2-ene, 2,3,7-trimethyloct-2-ene, 4,4,5-trimethylhex-2-ene, 3-methylhept-3-ene, 3,4-dimethylhex-2-ene, 2-ethylhex-1-ene, (E)-oct-3-ene, 2,4-dimethylhept-1-ene) and furans (furan, furan-3-carbaldehyde, 3-methylfuran, furan-2-carbaldehyde, 2-(methoxymethyl)furan) (Table 3 and Table 4). The first VOCs related to agar-agar cheese model boli were hydrophobic, i.e., with LogP (octanol/water) between 3.00 and 6.31. More hydrophilic VOCs were then released from agar-agar samples, such as furans, 1-propoxypropan-2-ol or 6-methylheptan-2-one. The release of these more hydrophilic VOCs could be related to the more hydrophobic character of agar-agar compared to κ-carrageenan. Indeed, agar-agar contains fewer hydrophilic sulfate groups than κ-carrageenan (1.1 ± 0.1% wt for agar-agar and 17.20 ± 0.5% wt for κ-carrageenan [62]) and is therefore more hydrophobic [63]. This less negatively charged and hydrophobic texturing agent could thus enable the release of hydrophilic VOCs.

Few VOCs (4-methylpent-3-ene-2-one, 1H-pyrrole) were associated with the carrageenan samples, i.e., few VOCs contributed negatively to the second component (Table 3 and Table 4). The VOCs of the cheese model boli seemed to have higher matrix affinity and thus higher retention and lower release with κ-carrageenan than with agar-agar. Working on structured guava bars formulated with agar-agar and low- and high-acyl gellan gum, da Costa et al. [46] observed that agar-agar allowed the release of a high number of VOCs responsible for the flavor of the product compared to gellan gum, both low and high acyl. Low-acyl gellan gum is known to produce a gel with a texture similar to that of agar-agar or ĸ-carrageenan [10]. Thus, the highest release of VOCs from products containing agar-agar observed by da Costa et al. [46] is in line with the highest release of VOCs from cheese models containing agar-agar in the present study. Κ-Carrageenan thus seemed to have a higher retention capacity compared to agar-agar. This finding is in good agreement with the fact that carrageenans are known to be efficient components for flavor encapsulation and immobilization by forming covalent and hydrogen bonds [47]. When working with ĸ-carrageenan and agar-agar gels, Zhao et al. [48] observed that the addition of κ-carrageenan to agar-agar gels increased the retention efficiency of the positively charged drug metformin hydrochloride (MET) and thus decreased its release. This phenomenon was mainly due to electrostatic interactions between the drug and the texturing agents. This finding is in line with that of our study, according to which κ-carrageenan seems to have a high retention capacity toward various compounds, including VOCs.

Therefore, chemical classes usually observed in dairy products such as lactones and ketones were associated with the unheated samples. Heating the samples for 20 min had an impact on the VOCs of the cheese models studied. Indeed, heating favored the generation and release of Maillard compounds and the volatilization of highly volatile compounds, as mentioned in the literature. The texturing agent type seemed to have an impact on flavor release. The addition of κ-carrageenan induced an increase in VOC retention, whereas agar-agar favored the release of greater amounts of VOCs. Thus, VOC analysis revealed that the texturing agent type and level, as well as heating, seemed to impact the VOC release from the processed cream cheese model boli at the swallowing point, which is consistent with the previous results of sensory analysis.

### 2.4. Prediction of the “Fresh Cream” Aroma Descriptor by the VOC Composition

The regression tree and random forest methods were performed to predict the “fresh cream” aroma sensory descriptor on the basis of its VOC characteristics. This descriptor was selected because it had the highest Fisher value (5.77, *p* < 0.05) in the two-way ANOVA (sensory descriptor = subject (random effect) + product (fixed effect)) on the aroma R-A-T-A results (Table 2). In addition, it is one of the main drivers of the liking of most soft cheeses [4].

**Figure 5:**
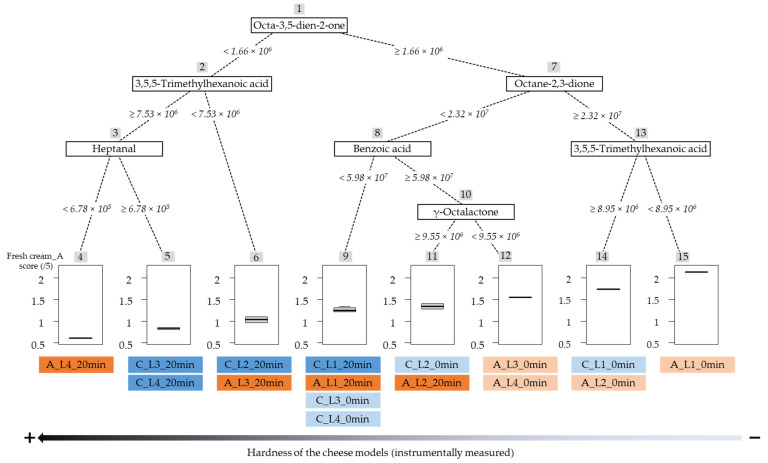
Decision tree (built with positively correlated VOCs) for “Fresh cream_A” sensory descriptor.

Although the cheese models studied contained numerous VOCs, certainly not all of them were expected to contribute as much to the odor and aroma perception. Indeed, it is known that only a small portion of VOCs present in foods are flavor-active [64]. The importance, i.e., the contribution, of VOCs as predictors of the “fresh cream” aroma descriptor is listed in Table 5. Among all VOCs, 18 contributed significantly to the “fresh cream” aroma prediction: 2 methyl ketones (octa-3,5-dien-2-one, acetophenone), 2 furans (2-methylfuran, 5-methylfuran-2-carbaldehyde), 4 diketones (octane-2,3-dione, pentane-2,3-dione, cyclohex-2-ene-1,4-dione, cyclopent-4-ene-1,3-dione), 5 aldehydes (heptanal, (E,E)-hepta-2,4-dienal, (E)-but-2-enal, 2-methylbutanal, 2-methylbut-2-enal), 2 acids (3,5,5-trimethylhexanoic acid, benzoic acid), 1 aromatic hydrocarbon (ethylbenzene) and 2 lactones (γ-octalactone, δ-decalactone). Among these 18 COVs, only those that had a positive impact on the “fresh cream” aroma were selected to build the decision tree represented in Figure 5.

As illustrated in Figure 5, six different VOCs (octa-3,5-dien-2-one, octane-2,3-dione, 3,5,5-trimethylhexanoic acid, heptanal, benzoic acid and γ-octalactone), each associated with a peak area threshold, allowed the splitting of the 16 cheese models of the experimental design according to their scores for the “fresh cream” aroma descriptor. The cheese models were divided into eight groups, from the hardest (instrumentally measured), with the lowest “fresh cream_a” score, to the softest, with the highest “fresh cream_a” score, from left to right.

The first VOC that allowed the whole set of cheeses to be split into two groups was “octa-3,5-dien-2-one” at an ion (*m*/*z* 95) peak area threshold value of 1.66 × 10^6^. Five cheese models, the hardest in terms of texture, with an octa-3,5-dien-2-one ion (*m*/*z* 95) peak area below this threshold, were classified together. The 11 remaining cheese models, with an octa-3,5-dien-2-one ion (*m*/*z* 95) peak area higher than 1.66 × 10^6^, were first split according to the octane-2,3-dione compound (threshold value of 2.32 × 10^7^). Eight cheese models, having medium hardness levels, with an octane-2,3-dione ion (*m*/*z* 99) peak area lower than the threshold value, were grouped together. The three remaining samples, softer in terms of texture, were finally divided according to the 3,5,5-trimethylhexanoic acid compound (threshold value of 8.95 × 10^6^). The highest score for the “fresh cream” aroma descriptor was obtained with a 3,5,5-trimethylhexanoic acid ion (*m*/*z* 57) peak area higher than the threshold value and for the product “A_L1_0min”. The highest scores for the “fresh cream” aroma descriptor were thus obtained by the softer cheese models (no heat treatment and low texturing agent level), which is coherent with the literature [2,4,38].

Thus, according to the RT and RF methodologies, high peak areas of octa-3,5-dien-2-one (*m*/*z* 95) and octane-2,3-dione (*m*/*z* 99) were needed for the highest “fresh cream” aroma perception in the cheese models studied, while a low 3,5,5-trimethylhexanoic acid peak area (*m*/*z* 57) was necessary. Octa-3,5-dien-2-one is known to be responsible for creamy, milky, cheesy and green aroma notes, and octane-2,3-dione is known for fatty, green and herbal ones (Table 5), which is consistent with a “fresh cream” aroma. It is worth noting that octa-3,5-dien-2-one has a low aroma detection threshold in water (0.150 ppm), which could explain its high contribution to the “fresh cream” aroma perception. Indeed, VOCs with low detection thresholds are frequently essential for aroma [54]. Our finding is in line with that of Bonaïti et al. [68], who found that octa-3,5-dien-2-one was a potent odor-active compound (dynamic headspace GC-O/MS identification) of Livarot cheese and cheese models. Moreover, Gallardo-Escamilla et al. [69] observed, while working on dairy products and performing PLS regression, that pentane-2,3-dione was among the VOCs that contributed the most to the prediction of “yogurt odor”. Pentane-2,3-dione belongs to the same chemical class as octa-3,5-dien-2-one and octane-2,3-dione, the ketones. Thus, ketones seem to play an important role in the odor and flavor of dairy products [67,68,69]. In addition to octa-3,5-dien-2-one, Bonaïti et al. [68] also observed that aldehydes such as (E,E)-hepta-2,4-dienal and heptanal were potent odorants of cheeses, which is consistent with our results, as these two aldehydes were among the VOCs with the highest V.I. While heptanal was positively linked to the “fresh cream” aroma perception in the present study, Kohama-Kobuchi et al. [4] found that this VOC was negatively linked (Pearson correlation) to the “yogurt aroma” of cheeses. The green and fatty notes of heptanal could explain its positive contribution to the “fresh cream” aroma and its negative impact on more acid aromas, such as yogurt.

In contrast to the volatile compounds mentioned above, VOCs such as 2-methylfuran (associated with cocoa, coffee and ethereal aroma notes), ethylbenzene (associated with metallic, phenolic and chemical notes) and 2-methylbutanal (associated with nutty, caramellic and cocoa notes) could mask the perception of the “fresh cream” aroma. In a study on cheddar cheese, Rizzo et al. [67] identified ethylbenzene as an aroma-active compound. Moreover, Bonaïti et al. [68] found that 2-methylbutanal and an aromatic hydrocarbon such as toluene were potent odorants of cheeses. Thus, the results of these two studies are consistent with our findings. The formation of these VOCs, mainly during the Maillard reaction for furans [41], should thus be limited in order to avoid their negative impact on the “fresh cream” aroma. This could be achieved by applying short heating times, for example, as the findings of this work showed that the unheated cheese models had the highest “fresh cream” aroma scores. The unheated cheese models were therefore less subject to the negative impact of 2-methylfuran, ethylbenzene or 2-methylbutanal.

Therefore, the regression tree and random forest methodologies enabled a better comprehension of how aroma perception was influenced by the presence or absence, to a certain extent, of key VOCs. Thanks to this statistical tool, it was possible to prioritize the importance of VOCs with the V.I. calculation and to identify a combination of VOCs (associated with threshold areas) that led to a given aroma perception. The highest scores for the “fresh cream” aroma descriptor were obtained by the cheese models with the highest areas of octa-3,5-dien-2-one and octane-2,3-dione, corresponding to the softer cheese models (no heat treatment and low texturing agent level). The increase in hardness with heating and the high level of the texturing agent could be responsible for the decrease in the release of endogenous VOCs such as ketones and the increase in the release of Maillard VOCs such as furans. These two phenomena could explain the decrease in the perception of the “fresh cream” aroma for firmer processed cream cheese models.

## 3. Materials and Methods

### 3.1. Materials

**Figure 6:**
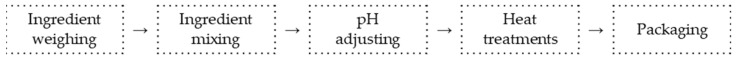
Optimized laboratory-scale production protocol of the cheese models.

The processed cream cheese models were composed of curd cheese, fresh cream, water, milk proteins, lactic ferments, salts, κ-carrageenan and agar-agar. They were produced at the laboratory scale (Bel, Vendôme, France) according to the protocol in Figure 6.

Cheese models were packed in plastic containers (115 ± 5 g) sealed with an aluminum lid. They were then stored at 4 °C until analysis. Artificial saliva was prepared weekly according to Van Ruth et al. [70] by dissolving, in 1 L of purified water (Milli-Q system, Millipore Corp., Molsheim, France), 0.44 g of CaCl_2_.2H_2_O (Merck, Darmstadt, Germany), 0.48 g of KCl (Merck, Darmstadt, Germany), 0.88 g of NaCl (Fluka, Steinheim, Germany), 1.37 g of K_2_HPO_4_.3H_2_O (Panreac, Barcelona, Spain), 2.16 g of porcine mucin (Sigma, St. Louis, MO, USA), 5.21 g of NaHCO_3_ (Merck, Darmstadt, Germany) and 13.00 g of porcine α-amylase (Sigma, St. Louis, MO, USA). N-Alkanes (C_5_–C_25_) of analytical grade and the standards used to identify the volatile compounds were purchased from Sigma Aldrich (St. Louis, MO, USA).

### 3.2. Experimental Design at Laboratory Scale

Sixteen processed cream cheese models were produced according to a three-factor experimental design (Table 1):

(1) Texturing agent type (2 levels): κ-carrageenan and agar-agar; these two components were chosen due to their different charges [12,13,62].
(2) Texturing agent level (4 levels): for κ-carrageenan cheese models: 1 = 0.20% (*w*/*w*), 2 = 0.35% (*w*/*w*), 3 = 0.50% (*w*/*w*), 4 = 0.65% (*w*/*w*); for agar-agar cheese models: 1 = 0.60% (*w*/*w*), 2 = 0.90% (*w*/*w*), 3 = 1.20% (*w*/*w*), 4 = 1.50% (*w*/*w*). Different contents were selected to produce processed cream cheese models comparable in hardness (instrumentally measured), regardless of the texturing agent type, for both unheated and heated samples (Figure 2). As κ-carrageenan is known to be more viscous (240 ± 2.0 mPa·s) than agar-agar (29 ± 2.0 mPa·s) [62], highest agar-agar quantities were needed to obtain cheese models with the same hardness as κ-carrageenan.
(3) Heating time (2 levels): 0 and 20 min.

Two formulas (C_L1_20min and A_L3_0min) were produced three times to study the variability in production at the laboratory scale. While the content of the texturing agent was variable, those of all of the other ingredients were constant. It is worth noting that the processed cream cheese models used in this study were produced to remain as close as possible to commercial processed cream cheeses, except for the texturing agent content.

### 3.3. In Vitro Mastication

The masticator used to mimic in vivo mastication was composed of a 375 mL container, a sintered circle to reproduce the human tongue, and a central plunger with variable compression and rotation speeds [71]. A PolyEtherEtherKetone (PEEK) flat jaw was fixed on the plunger to reproduce the human palate. The sample container was maintained at 36 ± 1 °C via hot silicone belts (Vulcanic SAS, Neuilly sur Marne, France). Two hose clamps were used to hermetically close the container. The following in vitro parameters were used to reproduce the adult cheese mastication until swallowing: 14 tongue-palate compressions, a rotation speed of 15 rpm, a cheese model quantity of 33 g (in the form of two cubes of 16.5 g (3 × 2.5 × 2 cm^3^) each) and a volume of artificial saliva ([70]) of 4.9 mL. The cheese model and the artificial saliva were introduced into the sample container in one go before the mastication process began. The same in vitro protocol was applied to all cheese models in the experimental design. After in vitro mastication, 5 g of bolus was transferred to a 20 mL vial for volatile compound analysis.

### 3.4. Texture Analysis

Texture measurements (back-extrusion type) were performed on unchewed cheese models, i.e., before in vitro mastication. The product (10 ± 1 g) was placed in a Petri dish (55 mm diameter). A cylindrical probe (35 mm diameter) was installed on the measurement cell (maximum capacity of 2 kN) of a traction–compression device (Instron 5544, Instron S.A., Norwood, MA, USA). With the use of the Petri dish lid (53 mm diameter), compression was applied at a speed of 0.2 mm·s^−1^ to flatten the cheese until it completely covered the Petri dish. Data were recorded using Merlin software (version 5.04., Instron S.A., Norwood, MA, USA). The final charge, namely, the hardness, was extracted from the data.

### 3.5. Sensory Analysis

#### 3.5.1. Ethics

The sensory test was conducted in accordance with the Declaration of Helsinki. All applicable institutional and governmental regulations concerning the ethical use of human volunteers were complied with during this research study. Written consent was obtained from the panelists after reading detailed information about the analysis. The sensory test performed in this study was approved by the ethics evaluation committee of the National Institute of Health and Medical Research (IRB00003888, IORG0003254, FWA00005831).

#### 3.5.2. Organization

A Home-Use Rate-All-That-Apply (R-A-T-A) test was performed on 59 consumers (35 recruited by Mérieux NutriSciences (Saint-Herblain, France) and 24 recruited by Oniris (Nantes, France); 26 women, 33 men; average age: 37.1 ± 13.0 years old). This test was carried out at home due to the COVID-19 pandemic. The microbiological quality of the samples was controlled before analysis by Eurofins Analytics (Nantes, France). For each analysis, a 15 g piece of the cheese model was presented in a 5 cL plastic cup (La Bovida, Paris, France) coded with a three-digit random number. The cups were closed with a plastic lid (La Bovida, Paris, France), which was removed before sample evaluation.

The generation of the texture (spoon and in-mouth) and odor–flavor descriptors was carried out by five subjects (three women, two men, average age: 34.6 ± 9.1 years old) familiar with sensory analysis during a one-hour session. During this session, the panelists tasted eight cheese models with extreme texture and flavor in order to discover the product space: four cheese models with level 1 of the texturing agent and four with level 4, both unheated and heated ones. Only the most cited descriptors were selected and then described by consensus. A list of 20 descriptors (8 of texture and 12 of odor/aroma/taste) was thus pre-generated (Table 2).

The R-A-T-A test was conducted by modality: texture (spoon (S) and in-mouth (M)) and odor (O)/aroma (A)/taste (T) were evaluated separately. For each modality, the panelists had to participate in one training session of 45 min and four evaluation sessions of 20 min. Thus, each panelist had to perform ten sessions (five by modality) in total to analyze the 20 cheeses in the experimental design. The principles of the sensory analysis and R-A-T-A test were explained to the panelists during the training session. References were used to get familiar with the descriptors, and exercises with the R-A-T-A scale were performed to get familiar with it. A five-point scale was used to evaluate the descriptors: very low, low, medium, high and very high. To avoid panelist fatigue, five products were evaluated during each evaluation session. The panelists were free to take as much of the 15 g product as they wished. Four sessions were necessary to assess the 20 cheese models, which were divided into four blocks (A, B, C and D) of 5 products. Half of the panelists evaluated texture first (block order: A-B-C-D) and odor/aroma/taste second (block order: C-D-A-B); the other half evaluated odor/aroma/taste first (block order: A-B-C-D) and texture second (block order: C-D-A-B). Mérieux NutriSciences panelists used an online questionnaire via RedJade software (version 3.0.0., Martinez, CA, USA), whereas Oniris panelists answered on a paper sheet. Within each block, the product serving position was randomized according to a William’s Latin square.

### 3.6. Volatile Compound Analysis

Volatile compounds were extracted using HS-SPME, followed by separation, identification and semi-quantitation using GC-(ToF)MS.

A quantity of 5 g of in vitro bolus was placed in a 20 mL glass vial closed with a screw cap equipped with a Teflon septum. The vials were stored until analysis at 10 °C in an autosampler cooling tray (MPS, Gerstel GmbH, Mülheim an der Ruhr, Germany). A 1 cm Carboxen-PolyDiMethylSiloxane fiber (Car-PDMS fiber, 85 μm; Supelco, Bellefonte, PA, USA) was used for HS-SPME extraction. The fiber was conditioned before analysis according to the manufacturer guidelines, i.e., by heating it in the GC injection port at 300 °C for 30 min. Equilibrium and extraction steps were both conducted at 45 °C for 30 min. The SPME fiber was then desorbed and maintained in the injection port at 260 °C for 4 min using the splitless mode. Volatile compounds were separated on a polar capillary column (DB-WAX, 30 m length × 0.25 mm internal diameter × 0.50 μm thickness, Agilent Technologies, Santa Clara, CA, USA). Hydrogen was used as the carrier gas at a constant flow rate of 1.5 mL·min^−1^. The column temperature was held at 35 °C for 5 min and then increased at a rate of 6 °C·min^−1^ to 240 °C, at which temperature the column was held for 11 min. The total run time was 50.17 min. The GC (7890B, Agilent Technologies, Santa Clara, CA, USA) was coupled to a time-of-flight mass spectrometer (LECO Pegasus^®^ BT ToF, St. Joseph, MI, USA). The transfer line and the ion source temperatures were 250 °C. The mass detector acquisition rate was 10 spectra·s^−1^ with an electronic ionization energy of −70 eV and a mass range from 39 to 400 *m*/*z*.

ChromaTOF software (version 5.51.06.0., LECO, St. Joseph, MI, USA) was used for identification and semi-quantification. After deconvolution, the volatile compounds were identified according to three criteria: comparison of their mass spectra with those of the Wiley11 NIST17 (National Institute of Standards and Technology) and internal libraries; comparison of their linear retention indexes, once determined with the use of alkanes (C_5_–C_25_), with those in the NIST WebBook for a polar column [27]; and comparison with those of the corresponding standards when the standards were available. The following parameters were used for identification: minimum signal/noise: 150; minimum stick count: 3; relative abundance threshold: 1; minimum similarity for matches: 600; and minimum similarity before hit assignment: 700. Semi-quantification was performed on the quantitative ion area of each detected peak, with the quantitative ion being selected among the four main ions of the molecule.

### 3.7. Statistical Treatments

All instrumental analyses (texture and VOCs) were carried out in triplicate. The statistical analyses of the sensory data were conducted on 20 cheeses: the 16 products in the experimental design and 2 production replicates of 2 formulas. The statistical analyses of the instrumental data were performed on 18 cheese models: the 16 products in the experimental design and 1 production replicate of 2 formulas. For texture measurements, a one-way ANOVA (Y (hardness) = texturing agent level (fixed effect)) and a three-way ANOVA with second-order interactions (Y (hardness) = texturing agent type (fixed effect) + texturing agent level (fixed effect) + heating time (fixed effect) + interactions) were performed. Concerning R-A-T-A data, a two-way ANOVA was performed (Y (sensory descriptor) = subject (random effect) + product (fixed effect)), and a CVA was carried out. For volatile compound data, a three-way ANOVA with second-order interactions (Y (ion peak area) = texturing agent type (fixed effect) + texturing agent level (fixed effect) + heating time (fixed effect) + interactions) and a standardized PCA were performed on the specific ion peak areas of the VOCs of the cheese model boli obtained after in vitro mastication. For all of the ANOVA analyses, the type III square sum values were taken, and an α risk of 5% was used; if significance was observed, a Least Significant Difference (LSD) post hoc test was performed. As previously described by Vigneau et al. and Cardinal et al. [72,73], regression tree and random forest methodologies were performed to predict aroma sensory perception (quantitative response) by VOCs (quantitative predictors) released at the swallowing point. ANOVAs were conducted with Statgraphics Centurion 19 software (Statpoint Technologies, Warrenton, USA), PCA was carried out with XLStat software (version 20, Addinsoft, Paris, France), and CVA and RF were performed with R software (version 4.1.1., R Core Team 2021, packages: rpart, partykit, randomForestSRC).

## 4. Conclusions

This work provides a better understanding of how sensory perception and volatile compound release are affected by both composition (texturing agent type and level) and process (heating time) factors when chewing processed cream cheese models. An experimental design was successfully set up to investigate the impact of these three factors. The R-A-T-A test proved to be appropriate to describe the sensory perceptions of the processed cream cheese models, while the instrumental method “in vitro mastication coupled with HS-SPME-GC-(ToF)MS” proved to be robust and efficient in obtaining a VOC fingerprint of in vitro processed cream cheese model boli.

The results obtained in this study provide information on the changes in flavor and texture caused by a reduction in the texturing agent. Moreover, agar-agar cheese models are perceived to have “milk” odor notes and favor the release of a large number of VOCs, whereas κ-carrageenan cheeses are perceived as having a “granular” and “brittle” texture and a “sour” and “salty” taste, and they display a high VOC retention capacity. Substituting ĸ-carrageenan, a controversial texturing agent due to its risk of intestinal inflammation, with agar-agar, a less controversial one, would lead to changes in sensory perception and volatile compound release. The type of texturing agent is therefore an important element to consider when modulating the texture and flavor of cheeses. Moreover, heating induces firmer cheese models, both instrumentally and sensory, and promotes Maillard VOCs, such as sulfurs, furans or Strecker aldehydes, responsible for “cooked” and “chemical” aroma perceptions. The results of this study indicate that a reduction in the texturing agent can be counterbalanced by an increase in heating time to preserve the desired flavor and texture. The in-depth knowledge concerning the behavior of different charge texturing agents and the impact of heating time on flavor and texture can provide valuable information for cheese manufacturers wishing to reduce the texturing agent content and/or thinking about substitutes.

Additionally, this study enables, for the first time to our knowledge, the prediction of cheese aroma perception from VOC release at the swallowing point with the use of regression tree and random forest methodologies. These machine-learning tools are thus powerful for investigating the VOCs that contribute the most to the aroma perception of a complex matrix, such as processed cream cheese. Octa-3,5-dien-2-one, associated with creamy, cheesy and green aroma notes, and octane-2,3-dione, associated with fatty, green and herbal ones, are the two main VOCs that contribute positively to the “fresh cream” aroma perception. However, the formation of 2-methylfuran, associated with cocoa, coffee and ethereal aroma notes, and ethylbenzene, associated with metallic, phenolic and chemical ones, should be limited to avoid their negative impact on the “fresh cream” aroma. The highest scores for the “fresh cream” aroma descriptor were obtained by the softer processed cream cheese models (no heat treatment and low texturing agent level). Therefore, these results provide some basis for controlling the sensory quality of processed cream cheeses by analyzing the key volatile compounds by GC-MS. An approach that combines sensory and instrumental flavor analysis with the random forest statistical tool has the advantage of being efficient and enabling rapid learning for an understanding of cheese flavor. Thus, it should be encouraged among cheese industries.

In the future, it may be useful to perform olfactometric analysis to confirm the key aroma-active VOCs highlighted in this study and their descriptors. In addition, recombination tests of the key VOCs could validate their impact on the perception of the “fresh cream” aroma [74]. Moreover, the other aroma perceptions such as “fresh cheese” and “chemical” could be investigated, as they significantly discriminated between the processed cream cheese models (*p*-values < 0.05).

## Figures and Tables

**Table 1:** Experimental design.

Product Name	Texturing Agent Type	Texturing Agent Level	Heating Time (min)
C_L1_0min	Carrageenan	1	0
C_L2_0min	Carrageenan	2	0
C_L3_0min	Carrageenan	3	0
C_L4_0min	Carrageenan	4	0
C_L1_20min	Carrageenan	1	20
C_L2_20min	Carrageenan	2	20
C_L3_20min	Carrageenan	3	20
C_L4_20min	Carrageenan	4	20
A_L1_0min	Agar-agar	1	0
A_L2_0min	Agar-agar	2	0
A_L3_0min	Agar-agar	3	0
A_L4_0min	Agar-agar	4	0
A_L1_20min	Agar-agar	1	20
A_L2_20min	Agar-agar	2	20
A_L3_20min	Agar-agar	3	20
A_L4_20min	Agar-agar	4	20

A: agar-agar samples (orange shading), C: carrageenan samples (blue shading); LX: level X of texturing agent; Heating time: 0 min (light shading)—20 min (dark shading).

**Table 2:** R-A-T-A sensory descriptor table and two-way ANOVA results (sensory descriptor = subject (random effect) + product (fixed effect)) for the “product” effect; α = 5%; * *p* < 0.05, *** *p* < 0.001. Shading—very light gray: texture descriptors (M: in-mouth; S: spoon); light gray: odor descriptors (O); dark gray: taste descriptors (T); very dark gray: aroma descriptors (A).

Descriptor	Evaluation Modality	Fisher	*p*-Value
Soft_M	Texture (in-mouth)	43.05	0.0000 ***
Firm_M	Texture (in-mouth)	38.83	0.0000 ***
Melting_M	Texture (in-mouth)	27.38	0.0000 ***
Sticky_S	Texture (spoon)	19.67	0.0000 ***
Brittle_S	Texture (spoon)	17.34	0.0000 ***
Rubbery_M	Texture (in-mouth)	10.07	0.0000 ***
Granular_M	Texture (in-mouth)	9.47	0.0000 ***
Salty_T	Taste	5.79	0.0000 ***
Fresh cream_A	Aroma	5.77	0.0000 ***
Tacky_M	Texture (in-mouth)	4.02	0.0000 ***
Fresh cheese_A	Aroma	5.19	0.0000 ***
Chemical_A	Aroma	4.92	0.0000 ***
Sour_T	Taste	2.44	0.0006 ***
Fermented_O	Odor	1.65	0.0392 *
Vinegar_O	Odor	1.35	0.1440
Fresh cheese_O	Odor	1.08	0.3648
Cooked_A	Aroma	1.05	0.4011
Butter_O	Odor	1.00	0.4537
Milk_O	Odor	0.65	0.8701
Milk_A	Aroma	0.60	0.9079

**Table 3:** List of VOCs identified in processed cream cheese models by HS-SPME-GC-(ToF)MS.

CAS Number	VOC Name	LRI_exp_	*m*/*z*	Stat.	CAS Number	VOC Name	LRI_exp_	*m*/*z*	Stat.
	**Acids**					**Alkanes**			
00064-19-7	Acetic acid	1455	43	u, -	00109-66-0	Pentane	499	43	h, -
00064-18-6	Formic acid	1517	46	u, -	00075-83-2	2,2-Dimethylbutane	516	43	-, -
00079-09-4	Propanoic acid	1548	45	u, a	00107-83-5	2-Methylpentane	550	43	-, -
00079-31-2	2-Methylpropanoic acid	1577	43	u, a	00096-14-0	3-Methylpentane	577	57	-, -
00075-98-9	2,2-Dimethylpropanoic acid	1587	57	-, -	00110-54-3	Hexane	598	57	h, -
00107-92-6	Butanoic acid	1636	60	u, -	01191-96-4	Ethylcyclopropane	628	42	-, -
00503-74-2	3-Methylbutanoic acid	1679	60	u, a	00096-37-7	Methylcyclopentane	682	56	h, -
00116-53-0	2-Methylbutanoic acid	1682	74	u, a	00142-82-5	Heptane	705	57	h, a
00109-52-4	Pentanoic acid	1749	60	u, -	00592-13-2	2,5-Dimethylhexane	715	57	-, a
03724-65-0	But-2-enoic acid	1786	86	u, c	00589-43-5	2,4-Dimethylhexane	719	43	-, a
00142-62-1	Hexanoic acid	1856	60	u, -	00110-82-7	Cyclohexane	725	56	-, -
00111-14-8	Heptanoic acid	1962	60	-, -	04516-69-2	1,1,3-Trimethylcyclopentane	732	55	-, a
03302-10-1	3,5,5-Trimethylhexanoic acid	1991	57	-, -	00592-27-8	2-Methylheptane	750	43	-, a
00124-07-2	Octanoic acid	2070	60	u, -	00589-53-7	4-Methylheptane	758	43	-, a
00110-44-1	*(2E,4E)-Hexa-2,4-dienoic acid*	2150	97	h, c	00589-81-1	3-Methylheptane	763	43	-, a
00112-05-0	Nonanoic acid	2177	60	-, -	02213-23-2	2,4-Dimethylheptane	805	43	-, a
00334-48-5	Decanoic acid	2283	60	u, -	03074-71-3	2,3-Dimethylheptane	845	43	-, a
14436-32-9	Dec-9-enoic acid	2347	55	u, -	02216-34-4	4-Methyloctane	850	43	-, a
00065-85-0	Benzoic acid	2459	105	u, -	15869-87-1	*2,2-Dimethyloctane*	894	57	h, a
00143-07-7	Dodecanoic acid	2496	73	u, -	62016-28-8	2,2,6-Trimethyloctane	932	57	h, a
	**Alcohols**				13475-82-6	2,2,4,6,6-Pentamethylheptane	951	57	h, a
00067-63-0	Propan-2-ol	943	45	-, c	62183-74-8	*2,2,3,3-Tetramethyloctane*	957	57	-, a
00064-17-5	Ethanol	949	45	h, -	17302-14-6	*2,2-Dimethylnonane*	970	57	-, a
00078-92-2	Butan-2-ol	1042	45	-, -	62016-30-2	2,3,3-Trimethyloctane	978	57	h, a
00071-23-8	Propan-1-ol	1057	42	h, c	62016-19-7	6-Ethyl-2-Methyloctane	1007	71	-, a
00077-74-7	*3-Methylpentan-3-ol*	1133	73	u, -	00124-18-5	Decane	1003	43	u, a
00071-36-3	Butan-1-ol	1166	56	-, -	01120-21-4	Undecane	1098	57	u, a
02566-44-1	2-Cyclopropylethanol	1176	67	u, -	04390-04-9	2,2,4,4,6,8,8-Heptamethylnonane	1250	57	u, -
00123-51-3	3-Methylbutan-1-ol	1220	55	-, -		**Aldehydes**			
00137-32-6	2-Methylbutan-1-ol	1224	57	-, -	00075-07-0	Acetaldehyde	708	44	h, a
01569-01-3	1-Propoxypropan-2-ol	1258	45	-, a	00123-38-6	Propanal	795	58	u, -
00763-32-6	3-Methylbut-3-en-1-ol	1264	41	-, -	00078-84-2	2-Methylpropanal	813	41	h, -
00071-41-0	Pentan-1-ol	1269	42	-, -	00123-72-8	Butanal	878	72	-, -
01576-96-1	*(E)-Pent-2-en-1-ol*	1361	57	u, a	00096-17-3	2-Methylbutanal	918	41	h, -
17540-75-9	2,6-Bis(1,1-dimethylethyl)-4-(1-methylpropyl)-phenol	1934	233	-, -	00590-86-3	3-Methylbutanal	922	44	h, -
00108-95-2	Phenol	2022	94	-, -	00123-73-9	(E)-But-2-enal	1051	70	h, -
00096-76-4	2,4-Di-t-butylphenol	2325	191	-, -	00066-25-1	Hexanal	1095	44	u, c
	**Alkenes**				01115-11-3	2-Methylbut-2-enal	1108	84	h, c
00590-18-1	(Z)-But-2-ene	511	41	h, a	01576-87-0	(E)-Pent-2-enal	1144	55	h, c
00504-60-9	Penta-1,3-diene	653	67	h, a	00111-71-7	Heptanal	1193	70	u, -
04050-45-7	*(E)-Hex-2-ene*	664	55	h, -	00107-86-8	3-Methylbut-2-enal	1212	84	-, -
00625-27-4	*2-Methylpent-2-ene*	677	69	-, -	55136-52-2	Pent-2-ynal	1227	53	h, c
00922-62-3	*(Z)-3-Methylpent-2-ene*	715	41	-, -	06728-26-3	(E)-Hex-2-enal	1232	41	-, c
02213-37-8	*3,4-Dimethylhex-2-ene*	772	83	-, a	20432-40-0	*(E,E)-Penta-2,4-dienal*	1243	81	h, a
01632-16-2	*2-Ethylhex-1-ene*	828	70	-, a	18829-55-5	(E)-Hept-2-enal	1339	83	u, -
14919-01-8	*(E)-Oct-3-ene*	840	41	-, a	00124-19-6	Nonanal	1405	56	u, -
07300-03-0	*3-Methylhept-3-ene*	841	83	-, a	00498-60-2	Furan-3-carbaldehyde	1441	96	-, a
55702-61-9	4,4,5-Trimethylhex-2-ene	858	83	-, a	02548-87-0	*(E)-Oct-2-enal*	1445	55	-, -
19549-87-2	2,4-Dimethylhept-1-ene	880	43	-, a	00098-01-1	Furan-2-carbaldehyde	1477	96	h, a
74421-06-0	5-Ethyl-2,4-dimethylhept-2-ene	996	83	-, a	04313-03-5	(E,E)-Hepta-2,4-dienal	1513	81	u, a
33933-75-4	*2,3,7-Trimethyloct-2-ene*	999	83	-, a	00100-52-7	Benzaldehyde	1543	77	u, a
06874-32-4	(Z) 3,7-Dimethyloct-2-ene	1020	70	-, a	00620-02-0	5-Methylfuran-2-carbaldehyde	1591	109	u, -
74421-03-7	2,4-Dimethyldec-2-ene	1078	83	-, a	00098-03-3	Thiophene-2-carboxaldehyde	1721	111	h, -
74630-52-7	(E)-6-Methylundec-3-ene	1174	57	-, -					
	**Ketones**					**Aromatic hydrocarbons**			
00431-03-8	Butane-2,3-dione	986	43	u, -	00071-43-2	Benzene	946	78	h, a
01629-58-9	*1-Penten-3-one*	1030	55	-, c	00108-88-3	Toluene	1050	91	-, a
00600-14-6	Pentane-2,3-dione	1073	43	u, a	00100-41-4	Ethylbenzene	1138	91	h, -
00585-25-1	Octane-2,3-dione	1332	99	u, -	00106-42-3	p-Xylene	1144	91	-, -
00930-30-3	Cyclopent-2-en-1-one	1374	82	-, c	00095-47-6	o-Xylene	1152	91	h, -
05704-20-1	2-Hydroxypentan-3-one	1376	45	u, a	00622-96-8	1-Ethyl-4-Methylbenzene	1237	105	-, -
01120-73-6	2-Methylcyclopent-2-en-1-one	1389	96	-, -	00100-42-5	Styrene	1270	104	-, -
13679-85-1	2-Methylthiolan-3-one	1551	60	h, -	00527-84-4	o-Cymene	1281	119	-, a
00930-60-9	Cyclopent-4-ene-1,3-dione	1605	54	h, -	00095-63-6	1,2,4-Trimethylbenzene	1294	105	-, -
04505-38-8	Cyclohex-2-ene-1,4-dione	1759	54	h, c	00091-20-3	Naphtalene	1769	128	u, -
00557-01-7	*Pyrimidin-2(1H)-one*	1796	96	h, -		**Furans**			
00067-71-0	Dimethyl sulfone	1923	79	u, -	00110-00-9	Furan	802	68	h, a
	**Methyl Ketones**				00534-22-5	*2-Methylfuran*	872	82	h, -
00067-64-1	Propan-2-one	816	43	h, a	00930-27-8	*3-Methylfuran*	902	82	h, a
00078-93-3	Butan-2-one	907	43	h, -	03208-16-0	2-Ethylfuran	961	81	h, a
00107-87-9	Pentan-2-one	984	43	h, -	03710-43-8	2,4-Dimethylfuran	973	96	h, a
00108-10-1	4-Methylpentan-2-one	1014	58	-, -	10504-04-8	2,3,5-Trimethylfuran	1068	109	h, a
00591-78-6	Hexan-2-one	1094	58	h, -	04466-24-4	2-Butylfuran	1143	81	h, a
00625-33-2	Pent-3-en-2-one	1139	69	-, -	03777-69-3	2-Pentylfuran	1242	81	h, a
00141-79-7	4-Methylpent-3-en-2-one	1145	98	h, c	13679-46-4	2-(Methoxymethyl)furan	1248	112	h, a
00110-43-0	Heptan-2-one	1194	43	h, -	13423-15-9	*3-Methyltetrahydrofuran*	1300	41	-, -
00928-68-7	6-Methylheptan-2-one	1247	58	-, a	00271-89-6	Benzofuran	1525	118	-, -
00111-13-7	Octan-2-one	1297	58	h, a	00098-00-0	2-Furanmethanol	1689	98	-, -
00513-86-0	3-Hydroxybutan-2-one	1302	45	-, -		**Nitrogen compounds**			
00110-93-0	6-Methylhept-5-en-2-one	1350	69	-, -	00075-05-8	Acetonitrile	1008	41	-, -
00821-55-6	Nonan-2-one	1401	43	h, -	00096-54-8	1-Methylpyrrole	1151	81	h, c
01669-44-9	Oct-3-en-2-one	1424	55	u, -	00290-37-9	Pyrazine	1228	80	-, a
00693-54-9	Decan-2-one	1507	58	h, a	02516-34-9	Cyclobutane-1-amine	1249	43	-, -
01192-62-7	1-(Furan-2-yl)ethanone	1520	95	h, a	00288-47-1	1,3-Thiazole	1265	85	h, -
38284-27-4	Octa-3,5-dien-2-one	1536	95	u, -	04786-24-7	3-Methylbut-2-enenitrile	1282	41	-, -
00112-12-9	Undecan-2-one	1613	58	h, -	01124-11-4	2,3,5,6-Tetramethyl pyrazine	1499	54	h, a
00098-86-2	Acetophenone	1675	105	u, -	00109-97-7	1H-Pyrrole	1529	67	h, c
00593-08-8	Tridecan-2-one	1826	58	-, -	00100-47-0	Benzonitrile	1627	103	-, -
	**Lactones**				04025-37-0	*2-(Aziridin-1-yl)ethanamine*	1641	44	u, -
00591-12-8	5-Methyl-3H-furan-2-one	1451	98	h, a		**Sulfur compounds**			
00096-48-0	Butyrolactone	1654	86	-, -	00074-93-1	Methanethiol	687	47	-, -
00591-11-7	2-Methyl-2H-furan-5-one	1703	55	h, -	00075-15-0	Carbon disulfide	729	76	h, a
00695-06-7	γ-Hexalactone	1730	85	u, -	00075-18-3	Dimethyl sulfide	748	47	-, a
00497-23-4	5H-Furan-2-one	1779	55	-, -	00624-92-0	Dimethyl disulfide	1085	94	-, -
00823-22-3	δ-Hexalactone	1825	42	u, -	00554-14-3	2-Methylthiophene	1103	97	h, c
00105-21-5	γ-Heptalactone	1836	85	u, -	03658-80-8	Dimethyl trisulfide	1399	47	-, -
00104-50-7	γ-Octalactone	1946	85	u, -		**Terpenes**			
00698-76-0	δ-Octalactone	2000	99	u, -	00080-56-8	α-Pinene	1026	93	h, a
00705-86-2	δ-Decalactone	2233	99	u, -	00127-91-3	β-Pinene	1114	93	-
00713-95-1	δ-Dodecalactone	2469	99	u, -	13466-78-9	3-Carene	1157	93	h, a
	**Esters**				05989-27-5	Limonene	1208	93	-, -
00141-78-6	Ethyl Acetate	895	43	h, a		**Unknown compounds**			
00105-54-4	Ethyl butanoate	1049	71	h, -	-	Unknown	988	57	-, -
01534-08-3	S-Methyl ethanethioate	1059	90	h, a	-	Unknown	997	57	-, -
00105-66-8	Propyl butanoate	1134	71	h, -	-	Unknown	1253	105	-, -
00123-66-0	Ethyl hexanoate	1244	88	-, a	-	Unknown	2159	97	-, -
04906-24-5	3-Oxobutan-2-yl acetate	1392	87	-, -					
03050-69-9	Vinyl hexanoate	1723	43	u, -					

The compounds are sorted by chemical class (bold text) and by increasing linear retention index_exp_ (LRI_exp_) within a single class. Italic text: uncertain identification; LRI_exp_: experimental linear retention index determined by injection of alkanes (C_5_–C_25_) on a DB-WAX column; *m*/*z*: quantitative ion selected among the four main ions; VOCs (tentatively) identified by comparing their mass spectra with the literature (Wiley11 NIST17 and internal libraries) and their LRI_exp_ with those reported in the NIST WebBook for a polar column and by confirming with standard references when available; “-”: not applicable; “Stat.”: ANOVA results (α = 5%) for “heating time” and “texturing agent type” factors. The different letters “h, u, c, a” indicate statistically significant differences, with VOCs more present in heated (h), unheated (u), carrageenan-based (c) and agar-agar-based (a) samples.

**Table 4:** Sixty main impacting VOCs at the swallowing point according to the results of dimensions 1 and 2 of PCA; the compounds are sorted from highest to lowest Variable Contributions (VCs); green shading: positive correlation; red shading: negative correlation.

Dim 1 (29.88%)				Dim 2 (19.04%)			
CAS Number	VOC Name	VC	Correlation	CAS Number	VOC Name	VC	Correlation
01534-08-3	S-Methyl ethanethioate	1.36	+0.94	02216-34-4	4-Methyloctane	2.17	+0.94
00695-06-7	γ-Hexalactone	1.32	−0.92	74421-06-0	5-Ethyl-2,4-dimethylhept-2-ene	2.17	+0.94
00698-76-0	δ-Octalactone	1.32	−0.92	74421-03-7	2,4-Dimethyldec-2-ene	2.15	+0.94
00104-50-7	γ-Octalactone	1.30	−0.92	02213-23-2	2,4-Dimethylheptane	2.14	+0.94
00504-60-9	Penta-1,3-diene	1.26	+0.90	03074-71-3	2,3-Dimethylheptane	2.08	+0.92
00096-17-3	2-Methylbutanal	1.25	+0.90	33933-75-4	2,3,7-Trimethyloct-2-ene	2.05	+0.92
00534-22-5	2-Methylfuran	1.25	+0.90	00589-81-1	3-Methylheptane	2.01	+0.91
00823-22-3	δ-Hexalactone	1.25	−0.90	55702-61-9	4,4,5-Trimethylhex-2-ene	1.98	+0.90
00071-43-2	Benzene	1.24	+0.90	00589-53-7	4-Methylheptane	1.98	+0.90
00288-47-1	1,3-Thiazole	1.23	+0.89	00592-13-2	2,5-Dimethylhexane	1.96	+0.90
00098-86-2	Acetophenone	1.23	−0.89	07300-03-0	3-Methylhept-3-ene	1.88	+0.88
04313-03-5	(E,E)-Hepta-2,4-dienal	1.21	−0.89	00589-43-5	2,4-Dimethylhexane	1.82	+0.87
00591-78-6	Hexan-2-one	1.20	+0.88	04516-69-2	1,1,3-Trimethylcyclopentane	1.81	+0.86
00123-73-9	(E)-But-2-enal	1.18	+0.87	02213-37-8	3,4-Dimethylhex-2-ene	1.80	+0.86
38284-27-4	Octa-3,5-dien-2-one	1.15	−0.86	01632-16-2	2-Ethylhex-1-ene	1.78	+0.86
00585-25-1	Octane-2,3-dione	1.15	−0.86	14919-01-8	(E)-Oct-3-ene	1.76	+0.85
03050-69-9	Vinyl hexanoate	1.13	−0.86	01120-21-4	Undecane	1.74	+0.85
00065-85-0	Benzoic acid	1.11	−0.85	19549-87-2	2,4-Dimethylhept-1-ene	1.72	+0.84
00107-87-9	Pentan-2-one	1.11	+0.85	00592-27-8	2-Methylheptane	1.54	+0.80
00109-66-0	Pentane	1.10	+0.84	00110-00-9	Furan	1.47	+0.78
00554-14-3	2-Methylthiophene	1.09	+0.84	00498-60-2	Furan-3-carbaldehyde	1.41	+0.76
00431-03-8	Butane-2,3-dione	1.08	−0.84	00124-18-5	Decane	1.39	+0.76
00110-43-0	Heptan-2-one	1.08	+0.84	00930-27-8	3-Methylfuran	1.37	+0.75
00064-19-7	Acetic acid	1.08	−0.84	01569-01-3	1-Propoxypropan-2-ol	1.31	+0.73
00590-18-1	(Z)-But-2-ene	1.06	+0.83	00098-01-1	Furan-2-carbaldehyde	1.27	+0.72
00107-92-6	Butanoic acid	1.05	−0.82	13679-46-4	2-(Methoxymethyl)furan	1.26	+0.72
04050-45-7	(E)-Hex-2-ene	1.05	+0.82	00928-68-7	6-Methylheptan-2-one	1.25	+0.72
01576-87-0	(E)-Pent-2-enal	1.04	+0.82	00096-14-0	3-Methylpentane	1.16	+0.69
00142-82-5	Heptane	1.02	+0.81	00141-79-7	4-Methylpent-3-ene-2-one	1.11	−0.68
00109-52-4	Pentanoic acid	1.00	−0.81	00109-97-7	1H-Pyrrole	1.06	−0.66

**Table 5:** Main Variable Importance (V.I.) of VOCs for “Fresh cream_A” descriptor and their aroma characteristics (green shading: positive correlation; red shading: negative correlation).

CAS Number	VOC Name	V.I.	Aroma/Flavor Description	Aroma Detection Threshold in Water (ppm)
38284-27-4	Octa-3,5-dien-2-one	3.58	Green ^1^, sweet ^2^, cooked ^2^, creamy ^2^, coconut ^2^, milky ^2^, cheesy ^2^	0.150 ^2^
00534-22-5	2-Methylfuran	3.13	Cocoa ^3^, ethereal ^3^, green ^3^, nutty ^3^, almond ^3^, coffee ^3^	-
00585-25-1	Octane-2,3-dione	3.10	Green ^3^, cilantro ^3^, fatty ^3^, leafy ^3^, herbal ^3^	-
00111-71-7	Heptanal	2.97	Green ^3^, oily ^3^, grassy ^3^, clover ^3^, cilantro ^3^	[0.003; 0.060] ^2^
04313-03-5	(E,E)-Hepta-2,4-dienal	2.93	Fatty ^3^, greasy ^3^, oily ^2^, green ^2^, herbal ^2^	-
03302-10-1	3,5,5-Trimethylhexanoic acid	2.51	-	-
00600-14-6	Pentane-2,3-dione	2.33	Toasted ^3^, buttery ^2^, fermented ^2^, dairy ^2^, creamy ^2^	0.020 ^2^
00123-73-9	(E)-But-2-enal	2.32	Plastic ^4^	-
00100-41-4	Ethylbenzene	2.30	Metallic ^5^, phenolic ^5^, chemical ^5^	-
00620-02-0	5-Methylfuran-2-carbaldehyde	2.24	Brown ^3^, sweet ^3^, caramellic ^3^, grain ^3^, maple ^3^	6.000 ^2^
00098-86-2	Acetophenone	2.14	Powdery ^3^, bitter almond ^3^, cherry ^3^	0.170 ^2^
00096-17-3	2-Methylbutanal	2.12	Fusel ^3^, nutty ^3^, caramellic ^3^, cocoa ^3^	-
00065-85-0	Benzoic acid	1.82	Pungent ^1^, sour ^1^	85.000 ^2^
00104-50-7	γ-Octalactone	1.58	Lactonic ^3^, coconut ^3^, creamy ^3^, sweet ^2^, fatty ^2^	0.007 ^2^
04505-38-8	Cyclohex-2-ene-1,4-dione	1.55	-	-
01115-11-3	2-Methylbut-2-enal	1.50	Fresh ^3^, fruity ^3^, green ^3^, almond ^3^, nutty ^3^	-
00705-86-2	δ-Decalactone	1.46	Coconut ^3^, creamy ^3^, fatty ^3^, buttery ^3^, milky ^3^	0.100 ^2^
00930-60-9	Cyclopent-4-ene-1,3-dione	1.44	Smoky ^5^, ashy ^5^	-

^1^ Aroma/flavor description from Flavor and Extract Manufacturer Association (https://www.femaflavor.org) (accessed on 20 September 2022) [65]; ^2^ aroma/flavor description and detection from *Fenaroli’s Handbook* [53]; ^3^ aroma/flavor description from http://www.thegoodscentscompany.com (accessed on 20 September 2022) [55]; ^4^ aroma/flavor description from Venkateshwarlu et al., 2004 [66]; ^5^ aroma/flavor description from Rizzo et al., 2022 [67]; “-”: data not found.

## Data Availability

Data are available on request due to restrictions, e.g., for privacy or ethical reasons.

## Sample Availability

Samples are not available from the authors.
